# Predicting progression of Alzheimer’s disease using blood-based multi-omics data

**DOI:** 10.1093/bioadv/vbag085

**Published:** 2026-03-28

**Authors:** Yashu Vashishath, Bizhan Alipour Pijani, Neha Goud Baddam, Fahad Saeed, Serdar Bozdag, for the Alzheimer’s Disease Neuroimaging Initiative

**Affiliations:** Department of Computer Science and Engineering, University of North Texas, Denton, TX 76203, United States; Center for Computational Life Sciences, University of North Texas, Denton, TX 76203, United States; BioDiscovery Institute, University of North Texas, Denton, TX 76203, United States; Department of Computer Science and Engineering, University of North Texas, Denton, TX 76203, United States; Center for Computational Life Sciences, University of North Texas, Denton, TX 76203, United States; BioDiscovery Institute, University of North Texas, Denton, TX 76203, United States; Department of Computer Science and Engineering, University of North Texas, Denton, TX 76203, United States; Center for Computational Life Sciences, University of North Texas, Denton, TX 76203, United States; BioDiscovery Institute, University of North Texas, Denton, TX 76203, United States; School of Computing and Information Sciences, Florida International University, Miami, FL 33199, United States; Department of Computer Science and Engineering, University of North Texas, Denton, TX 76203, United States; Center for Computational Life Sciences, University of North Texas, Denton, TX 76203, United States; BioDiscovery Institute, University of North Texas, Denton, TX 76203, United States; Department of Mathematics, University of North Texas, Denton, TX 76203, United States

## Abstract

**Motivation:**

Early prediction of Alzheimer’s disease (AD) progression from mild cognitive impairment (MCI) remains a major challenge, particularly when relying on non-invasive biomarkers. Identifying individuals with progressive MCI (pMCI) before conversion to AD could improve intervention strategies and clinical management. In this study, we developed a machine learning (ML) framework integrating blood-based multi-omics and demographic data to distinguish pMCI from stable MCI (sMCI).

**Results:**

We trained ML models using combinations of single nucleotide polymorphism (SNP), DNA methylation, gene expression, lipid, and bile acid metabolite data using both early and late data integration strategies. Late integration consistently outperformed early integration, with the L1-regularized logistic regression model achieving the highest F1 score of 90.7% when combining SNP and lipid data. Feature interpretability analyses using LIME and SHAP identified reproducible biomarkers across omic layers, including SNPs in MYH11, FOXP1, MAPK10, and SYN3; methylation changes in CDX2 and DHX58; and altered ceramide lipid CER.D19.1.24.0 levels, all previously associated with AD-related pathology. These findings demonstrate that combining multi-omics and demographic data can improve early prediction of AD progression and support the feasibility of blood-based, interpretable biomarkers for precision diagnostics.

**Availability and implementation:**

The data analyzed in this study were obtained from the Alzheimer’s Disease Neuroimaging Initiative (ADNI) database (https://adni.loni.usc.edu/) and are available to qualified researchers upon application. The code used in this study, together with instructions and a toy dataset, is available at: https://github.com/bozdaglab/AD_sMCI_vs_pMCI.

## Introduction

Alzheimer’s Disease (AD) is clinically characterized by a wide spectrum of dementia symptoms, including memory impairment, aphasia, speech dysfluency, visuospatial deficits, executive dysfunction, and personality or behavioral changes, the underlying cause of which remains unknown (Alzheimer’s Association 2019). As the most common cause of dementia, AD affects an estimated 30 million people worldwide and accounts for approximately 60%–80% of all dementia cases (Barker *et al.* 2002). Current treatments can only provide temporary relief for memory and cognitive problems but do not cure the disease (Alzheimer’s Association 2019). The failure of numerous AD drug trials is largely attributed to the inclusion of participants in the advanced stages of the disease (Cummings *et al.* 2014). Patients with Mild Cognitive Impairment (MCI), an early stage of AD, progress to the disease at an estimated rate of 10%–15% per year, with about 80% converting to AD within 6 years of follow-up (Alzheimer’s Association 2019). As explained in previous studies, if a patient remains in the MCI stage for an extended period, the diagnosis is considered stable MCI (sMCI); however, if conversion occurs within a 3 year period, it is classified as progressive MCI (pMCI) (Thung *et al.* 2016, Lu *et al.* 2022, Zhang *et al.* 2024). Early diagnosis plays a crucial role in improving patient care and may help delay or even prevent the onset of AD.

Recent advances in high-throughput technologies have enabled comprehensive profiling across multiple biological layers such as genomics, epigenomics, transcriptomics, and metabolomics, which are collectively referred to as multi-omics data. Multi-omics approaches offer unprecedented insights into the molecular mechanisms underlying complex diseases such as AD (Hasin *et al.* 2017). Integration of these heterogeneous data types has shown strong potential for improving predictive modeling and biomarker discovery for early-stage diagnosis (Zhavoronkov *et al.* 2019, Ren *et al.* 2025). Among these omics layers, five modalities have demonstrated particular relevance to AD pathology: gene expression, Single Nucleotide Polymorphism (SNP) from Genome-Wide Association Studies (GWAS), DNA methylation patterns, lipid metabolite profiles, and bile acid metabolites (Baloni *et al.* 2020). Incorporating demographic and clinical variables alongside these molecular features further enhances the ability to model disease heterogeneity among individuals.

A major focus of current AD research is distinguishing sMCI from pMCI, as this transition is a critical window for intervention (Singh *et al.* 2024). As summarized by Singh *et al.*, many studies apply Machine Learning (ML) to neuroimaging, clinical, or mixed datasets, using models that range from logistic regression and Support Vector Machine (SVM) to deep learning (Zhang and Shen 2019, Singh *et al.* 2024). Despite this progress, three gaps remain. First, much of the literature relies on imaging, which can be costly and less accessible for routine screening (Singh *et al.* 2024). Second, many approaches use a single data type or a fixed set of modalities, leaving open which combinations are most informative (Hasin *et al.* 2017, Zhavoronkov *et al.* 2019, Ren *et al.* 2025). Third, integration choices are often *ad hoc* even though the strategy for combining modalities can strongly affect performance (Huang *et al.* 2017).

**Figure 1:**
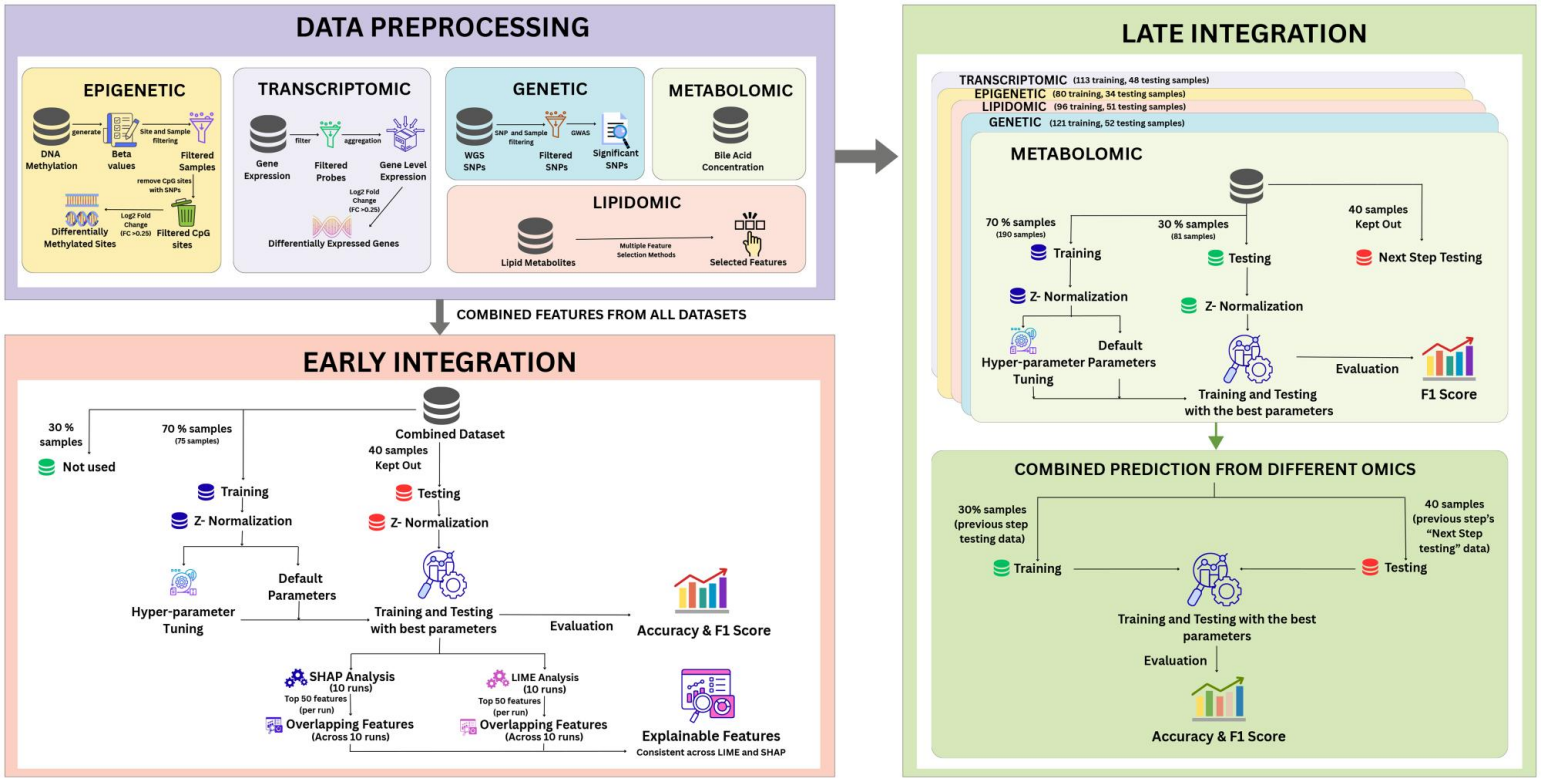
Overview of the multi-omics integration and predictive modeling pipeline. The study incorporates five omics data types—genomics (WGS SNPs), transcriptomics (gene expression), epigenomics (DNA methylation), lipidomics (lipid metabolites), and metabolomics (bile acid concentrations)—alongside demographic variables (age, gender, education, race, and ethnicity). Each omics layer underwent domain-specific preprocessing and feature selection (e.g. GWAS for SNPs, differential expression for gene expression and methylation). Two modeling strategies were employed: early integration, where all features were combined into a single dataset, and late integration, where separate models were trained on each modality and their outputs combined. ML classifiers were trained, tuned, and evaluated using accuracy and F1 score, with SHAP-based analysis performed for interpretability.

To address these gaps, we present a blood-based multi-omics framework designed to systematically compare early and late data integration strategies for predicting conversion from MCI to AD. Our framework integrates five omics modalities (SNPs, DNA methylation, gene expression, lipids, and bile acids) together with demographic features (Baloni *et al.* 2020). We comprehensively evaluate all modality combinations using five different ML classifiers: L1-regularized logistic regression (L1), SVM, Random Forest (RF), eXtreme Gradient Boosting (XGBoost), and Neural Network (NN), with performance assessed on a held-out test set (Fig. 1).

Our results demonstrate that the integration strategy substantially influences predictive performance. Late integration consistently outperforms early integration when all modalities are combined, indicating that independent model training followed by prediction-level fusion provides greater flexibility. Moreover, selective and low-cost modality subsets can achieve performance exceeding that of models trained on expensively obtained data types. Notably, the L1 model combining SNP and lipid features achieves an F1-score of 90.7% on the test set, comparing favorably with models reviewed by Singh *et al.* (Singh *et al.* 2024) while relying solely on peripheral biomarkers. To enhance interpretability, we apply Local Interpretable Model-Agnostic Explanations (LIME) (Ribeiro *et al.* 2016) and Shapley Additive Explanations (SHAP) (Lundberg and Lee 2017) to identify reproducible and biologically meaningful features associated with conversion risk. Overall, our study demonstrates that careful selection of integration strategy and omic modality can significantly improve early prediction of AD progression and supports the development of practical, interpretable tools for precision neurology.

## Methods

Data used in the preparation of this article were obtained from the Alzheimer’s Disease Neuroimaging Initiative (ADNI) database (http://adni.loni.usc.edu). The ADNI was launched in 2003 as a public-private partnership, led by Principal Investigator Michael W. Weiner, MD. The primary goal of ADNI has been to test whether serial magnetic resonance imaging (MRI), positron emission tomography (PET), other biological markers, and clinical and neuropsychological assessment can be combined to measure the progression of mild cognitive impairment (MCI) and early Alzheimer’s disease (AD). Our study incorporated different omics containing Whole Genome Sequencing (WGS), DNA methylation, gene expression, lipid metabolites, and bile acid metabolites.

### Diagnosis label

We collected patient visit information and aligned each individual with the corresponding diagnostic tags. Diagnostic labels were obtained from the *DX* (diagnosis) field in the ADNI dataset, which provides standardized clinical assessments for each visit. Participants who were initially diagnosed with MCI were monitored longitudinally for conversion to AD. As previously described, individuals who converted to AD within three years of their initial MCI diagnosis were labeled as pMCI, whereas those who remained stable for at least three years were classified as sMCI (Thung *et al.* 2016, Lu *et al.* 2022, Zhang *et al.* 2024). Data analysis was performed using R (R Core Team 2022), version 4.2.2.

### Sample details

We identified 115 samples containing all omics data modalities. Additionally, 98, 39, 86, 62, and 196 samples contained SNP, DNA methylation, gene expression, lipid, and bile acid data, respectively, while missing at least one other modality. To prevent data leakage during model development, 40 samples common across all data modalities were designated as a held-out test set and excluded from normalization, feature selection, and model training. For individual models, all available samples for each omics type were utilized independently. Table 1 shows counts of samples used for training and testing in early and late integration of modalities.

**Table 1:** Sample counts by diagnosis group (pMCI vs. sMCI) used in early and late integration. Train and initial test sets are pooled.

Modality	**Train + initial test**			**Final test**		
	Total	pMCI	sMCI	Total	pMCI	sMCI
**Early integration**						
All modalities	75	40	35	40	24	16
**Late integration**						
Genetic	173	100	73	40	24	16
Epigenetic	114	63	51	40	24	16
Transcriptomic	161	92	69	40	24	16
Lipidomic	137	77	60	40	24	16
Metabolomic	271	172	99	40	24	16

### SNP identification using GWAS

DNA isolation and SNP genotyping process has been described previously (Shen *et al.* 2010). We processed the genetic data by merging SNP information of all patients into a single set of files using PLINK software package (Purcell *et al.* 2007), release v1.06. The same software was also used for the GWAS analysis. The following criteria were used for SNPs filtering: missing call rate less than 90% were removed, Minor Allele Frequency (MAF) less than 5% were removed, and Hardy–Weinberg equilibrium test of p-value more than $10^{-6}$ were removed. Samples were also filtered by removing samples with a call rate less than 95%. SNPs with *P*-value $\leq 10^{-5}$ were selected, resulting in 84 features for downstream analysis.

### DNA methylation data pre-processing

DNA methylation data processing was conducted using the Minfi (Aryee *et al.* 2014) package in R/Bioconductor (R Core Team 2022), starting with raw intensity data from.idat files (*.Red.idat* and. *Grn.idat*). The raw data were loaded using *read.metharray.exp()* function to generate a rgSet object, from which detection *P*-values were calculated to assess the data quality. Samples with an average detection *P*-value > .05 were excluded, and probes failing in any sample or containing SNPs at CpG sites were removed. The remaining samples underwent quantile normalization using *preprocessQuantile()* function, resulting in a final dataset of approximately 684 000 probes. From this processed data, beta values (β-values) were computed using *getBeta()* function, which represent the ratio of methylated probe intensity (*M*) to the total signal intensity (i.e. sum of methylated and unmethylated (*U*)) (Equation 1).


(1) $$\beta =\frac{M}{M+U}$$

Methylation data was grouped by diagnosis (sMCI vs. pMCI), and the mean methylation value for each CpG site was calculated per group. Log_2_ fold change (log_2_FC) was computed using a small pseudocount ($\varepsilon =1\times 10^{-6}$) to avoid division by zero. CpG sites with an absolute log_2_FC ≥0.25 were selected for downstream analysis, resulting in 1584 DNA methylation features.

### Gene expression data pre-processing

ADNI provided preprocessed data for 49 293 trascript probes (Saykin *et al.* 2015). Expression values were extracted by removing control probes and aggregating multiple probes per gene using the maximum expression value among probes for a given gene. Samples without a clinical diagnosis label were excluded. Expression data was grouped by diagnosis (sMCI vs. pMCI), and the mean expression value for each gene was calculated per group. Log_2_FC was computed using a small pseudocount ($\varepsilon =1\times 10^{-6}$) to avoid division by zero. Genes with an absolute log_2_FC ≥0.25 were selected for downstream analysis, resulting in 173 features.

### Lipid data pre-processing

The tabular data were obtained from the ADNI portal under the filename *ADMCLIPIDOMICSMEIKLELABLONG.csv* (Huynh *et al.* 2020). The data were filtered to use the closest visit information for patients, consistent with the other omics datasets. Feature selection was based on a combination of filter-based, wrapper-based, and embedded techniques. The filter-based approach employed Mutual Information (MI) to quantify the statistical dependence between each feature and the target label; only features with positive MI scores were retained. Across all features, MI values ranged from 0 to 0.14, and features with an MI score of 0 were excluded. For the wrapper-based approach, we used a RF model with optimized hyperparameters to evaluate subsets of features via several strategies. Recursive Feature Elimination (RFE) iteratively removed the least-important features based on RF importance scores. *Select_From_Model* retained features with the highest importance weights, while *Select_By_Single_Feature_Performance* evaluated each feature individually using Area Under Curve (AUC), retaining those with AUC > 0.6. SHAP (Lundberg and Lee 2017) values were also computed to ank features according to their contributions to model predictions. In the embedded method, we applied LASSO regression on only training samples, which imposes an L1 penalty that drives coefficients of less-informative features to zero. The regularization parameter λ was tuned via cross-validation over a grid, and the model was refit 50 times; only features consistently selected across all runs were retained, ensuring stability across different λ settings. To increase the robustness of the selected feature set and reduce the risk of method-specific artifacts, we prioritized features that were selected by at least three of the six feature selection methods (i.e. MI, RFE, SelectFromModel, SelectBySingleFeaturePerformance, SHAP, and LASSO), resulting in a final set of 88 features.

### Bile acid data

The data was downloaded in a tabular format from ADNI portal with filename *ADMC_BA_POSTPROC.csv*, which was processed previously (John-Williams *et al.* 2017, MahmoudianDehkordi *et al.* 2019). All the 23 bile acid metabolite features were used in our predictive models.

### Multi-Omics data integration

We employed two common approaches for integrating multi-omics data in predictive models:

1. Early integration: In the early integration approach, features from all available omics datasets were merged into a single unified dataset. This combined dataset, containing all 1,956 features across different modalities including demographics, was then used as input to train ML models to predict sMCI vs. pMCI label for held-out test samples.
2. Late integration: In the late integration approach, we trained an independent ML model for each omics type. The complete data were divided into three parts: (i) training data for omics-specific models, which included samples that have data for the corresponding omics type even though they did not have data for another omics type. (ii) test data to test the initial models and train the meta learner. For this purpose 30% of the samples with complete data across all modalities were reserved. The prediction outputs from these common samples were then combined and used as input features to train a meta-learner. (iii) an independent set of 40 held-out samples to evaluate the performance of the integrated model. To ensure a fair comparison, the same 40 held-out samples were used to evaluate the early integration models, too.

### Model training and feature importance analysis

We employed five ML models, namely L1, SVM, RF, XGBoost, and NN, to predict the pMCI vs. sMCI label for a patient. Each model was executed ten times using three-fold cross-validation for hyperparameter tuning, and grid search was applied to identify the optimal configuration for final evaluation. For early integration, models were trained on all possible combinations of the five omics modalities and demographic features, resulting in a total of 31 models. For late integration, models were trained separately for each combination of multiple modalities (excluding single-modality cases), yielding 26 models in total.

To identify the most informative and reproducible features, we applied LIME (Ribeiro *et al.* 2016) and SHAP across datasets for the L1-regularized model, which demonstrated the best predictive performance. Feature importance estimation was repeated ten times, and the top 50 ranked features from each run were retained. Features consistently appearing across all ten runs were identified by computing the intersection of the ranked lists, yielding a consensus set of stable predictors. Features overlapping between LIME- and SHAP-derived selections were subsequently evaluated for statistically significant differences between sMCI and pMCI groups. Significance testing for continuous variables (lipid abundances and CpG methylation levels) was performed using Welch’s two-sample *t*-tests comparing pMCI and sMCI groups. For SNPs, we first tested the association between genotype (coded as 0, 1, or 2 copies of the minor allele) and diagnosis (pMCI vs. sMCI) using chi-square tests of independence on 3 × 2 contingency tables, obtaining one p-value per variant. We then estimated per-allele Odds Ratios (OR) and 95% confidence intervals using logistic regression with diagnosis as the binary outcome and SNP dosage as a continuous predictor; in this additive model, the OR represents the multiplicative change in the odds of progression to pMCI for each additional minor allele (OR >1 indicates increased risk, OR <1 indicates a potentially protective effect). Multiple hypothesis testing was controlled using the Benjamini–Hochberg False Discovery Rate (FDR), and statistical significance was reported using FDR-adjusted *P*-values. All analyses were conducted in Python 3.11 using NumPy (Harris *et al.* 2020), pandas (McKinney 2010), SciPy (Virtanen *et al.* 2020), scikit-learn (Pedregosa *et al.* 2011), Matplotlib (Hunter 2007), and Jupyter Notebook (Kluyver *et al.* 2016).

## Results

This section presents the results of model evaluation across different data integration strategies and omics combinations for distinguishing sMCI from pMCI patients. Model performance was compared under early and late integration frameworks using five different classifiers, namely L1, SVM, RF, XGBoost, and NN. In addition to overall classification performance, we analyzed feature importance and model interpretability to identify biomarkers that differ significantly between sMCI and pMCI conditions, providing insights into potential molecular mechanisms associated with disease progression.

### Model performance across integration strategies

**Figure 2:**
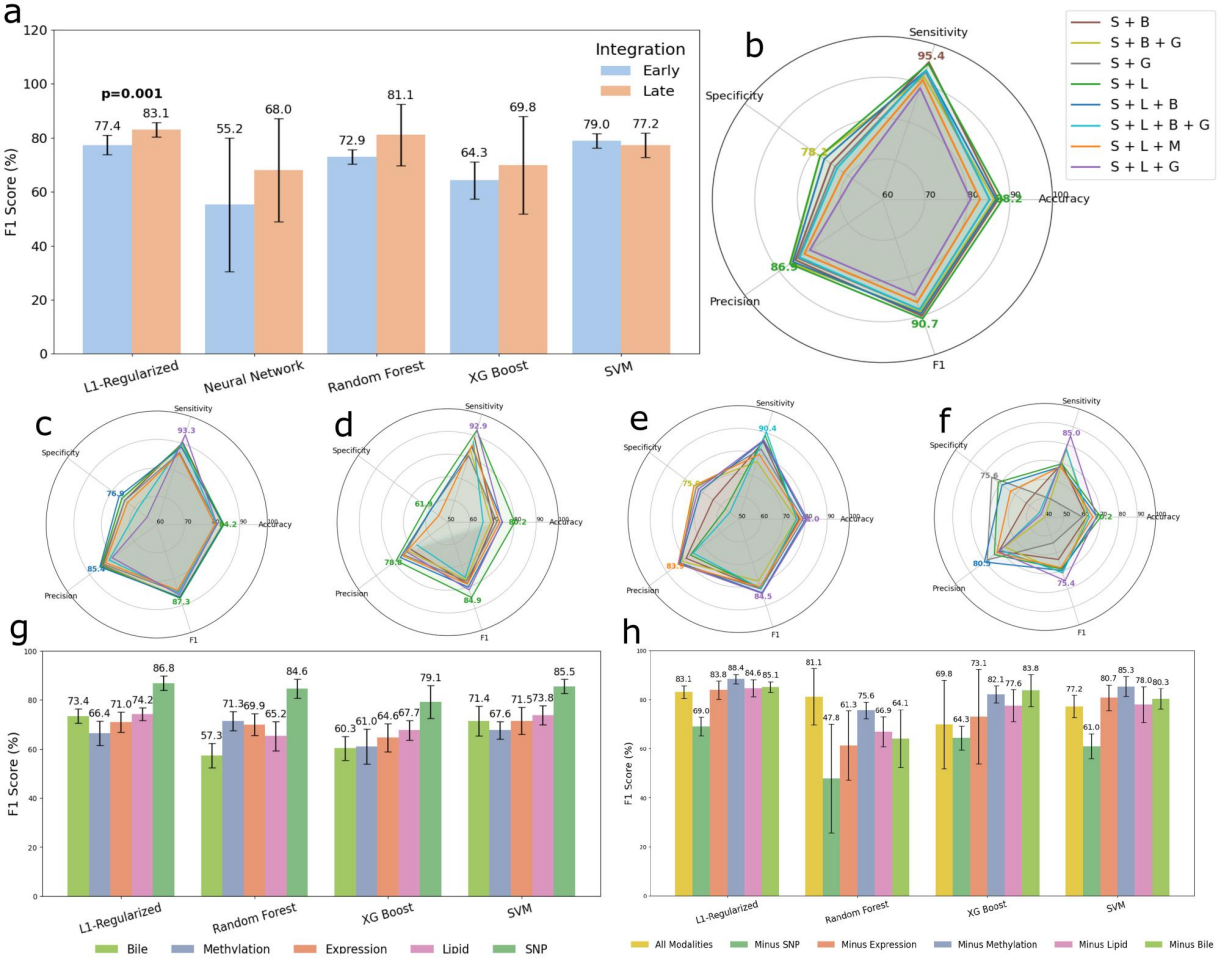
Model performance across data integration strategies and modalities. (a) Bar chart showing F1 score comparison between early and late integration strategies across five ML models using all data modalities combined while showing significant difference. (b–f) Radar plots illustrating performance metrics (accuracy, specificity, sensitivity, precision, and F1 score) for the best-performing combinations of data modalities using (b) L1-regularized logistic regression, (c) support vector machine, (d) random forest, (e) eXtreme Gradient Boosting and (f) neural network. (g) Bar chart showing F1 score performance for single-modality models across four algorithms (excluding NN due to high standard deviation). (h) Bar chart showing F1 score for late integration models when one data modality is removed (again excluding NN due to high standard deviation); “All modalities” represents the complete late integration setup. In radar plot legends, S denotes SNP, D denotes DNA methylation, G denotes gene expression, L denotes lipid, and B denotes bile acid modalities.

Figure 2 summarizes the comparative performance of the proposed models under early and late data integration strategies. As shown in Fig. 2a, late integration outperformed early integration for the L1, NN, RF, and XGBoost models when all omics modalities were combined, whereas early integration provided superior performance for the SVM model. The performance difference reached statistical significance only for the L1-regularized model. Overall, these findings indicate that modeling each omics layer independently and combining their predictions at a later stage tends to produce more expressive and informative representations than training a single model on concatenated multi-omics features. Among the models, the L1 model also achieved the highest F1 score, demonstrating strong predictive performance and robustness across all modalities combination.

In addition to comparing classifier performance, our framework systematically evaluated all possible combinations of omics modalities, enabling the identification of modality subsets that yielded the best predictive outcomes for each model (Supplementary Tables 1–5). Radar plots in Fig. 2b–f illustrate the best-performing modality combinations for each classifier, evaluated using accuracy, specificity, sensitivity, precision, and F1 score. The radar plot of L1 (Fig. 2b), SVM (Fig. 2c), and RF (Fig. 2d) show the best performance based on F1 score, was achieved when combining SNP (S) and Lipid (L) modalities, indicating that genetic and metabolomic features together provide strong discriminative power for predicting MCI progression. In contrast, the XGBoost model (Fig. 2e) performed best when S, L, and Gene expression (G) data were combined, suggesting that the addition of transcriptomic information further enhanced predictive capability. Neural network (Fig. 2f) results were inconsistent due to instability and high variance, likely caused by the limited sample size. Figure 2b–f also show that specificity was consistently lower than sensitivity, precision, and accuracy. This pattern is likely driven by a higher rate of false-positive predictions, potentially influenced by the greater number of pMCI cases relative to sMCI in the dataset (Table 1).

Figure 2g presents model performance using individual omics modalities. Among single modalities, S alone achieved the strongest F1-score and outperformed all other individual feature groups. However, multimodal combinations such as S + L for L1, SVM, and RF, and S + L+G for XGBoost surpassed the SNP-only model, indicating that selective feature integration can provide complementary information beyond that captured by genetics alone. For the L1 model in particular, the S + L combination yielded a significantly higher F1-score than S alone (*P*-value ≈0.009), demonstrating that lipid features meaningfully enhance predictive performance rather than merely contributing redundant signal.

Figure 2h shows the results of the leave-one-modality-out analysis under the late integration framework. For the L1, SVM, and XGBoost models, removing any modality except S resulted in improved performance compared with the “Minus None” configuration (i.e. utilizing all modalities), suggesting that certain modalities introduced redundant or noisy information. In contrast, the RF model exhibited the opposite trend of decreased performance whenever a modality was excluded, with the highest F1 score (81.1%) observed when all five modalities were combined. Across models, removal of the DNA methylation (D) modality generally led to slight performance gains; however, interpretability analyses using LIME and SHAP revealed several methylation features that were significantly different between sMCI and pMCI (see Section Feature Importance and Biomarker Identification. This suggests that while inclusion of DNA methylation data may introduce variability at the model level, it still captures biologically relevant signals associated with disease progression.

### Comparative evaluation

Overall, the proposed late integration framework achieved superior predictive performance compared to that reported for previously published approaches for forecasting MCI-to-AD progression. Among the evaluated models, the L1 model achieved the highest F1 score of 90.7% on the test data when combining SNP and lipid modalities (Fig. 2b), demonstrating strong predictive capability. Compared with recent studies that rely primarily on neuroimaging or cognitive assessment data, such as Liu *et al.* (Liu *et al.* 2017) and Liu *et al.* (Liu *et al.* 2023), our model achieved notably higher performance using only peripheral blood biomarkers. Specifically, Liu *et al.* (Liu *et al.* 2023) reported an adjusted F-measure of 73.41% using a novelty detection framework based on ApoE4 and cognitive-functional assessments, whereas Liu *et al.* (2017) achieved 84.62% accuracy using multimodal MRI and PET data. In contrast, our L1 model showed better performance while using low-cost, non-invasive omics data, underscoring the potential of blood-based biomarkers combined with interpretable ML for early AD prediction. This comparison is meant to provide contextual reference rather than a direct head-to-head evaluation, serving to illustrate that peripheral blood–derived features are capable of supporting competitive or superior predictive performance relative to more resource-intensive modalities.

### Feature importance and biomarker identification

**Figure 3:**
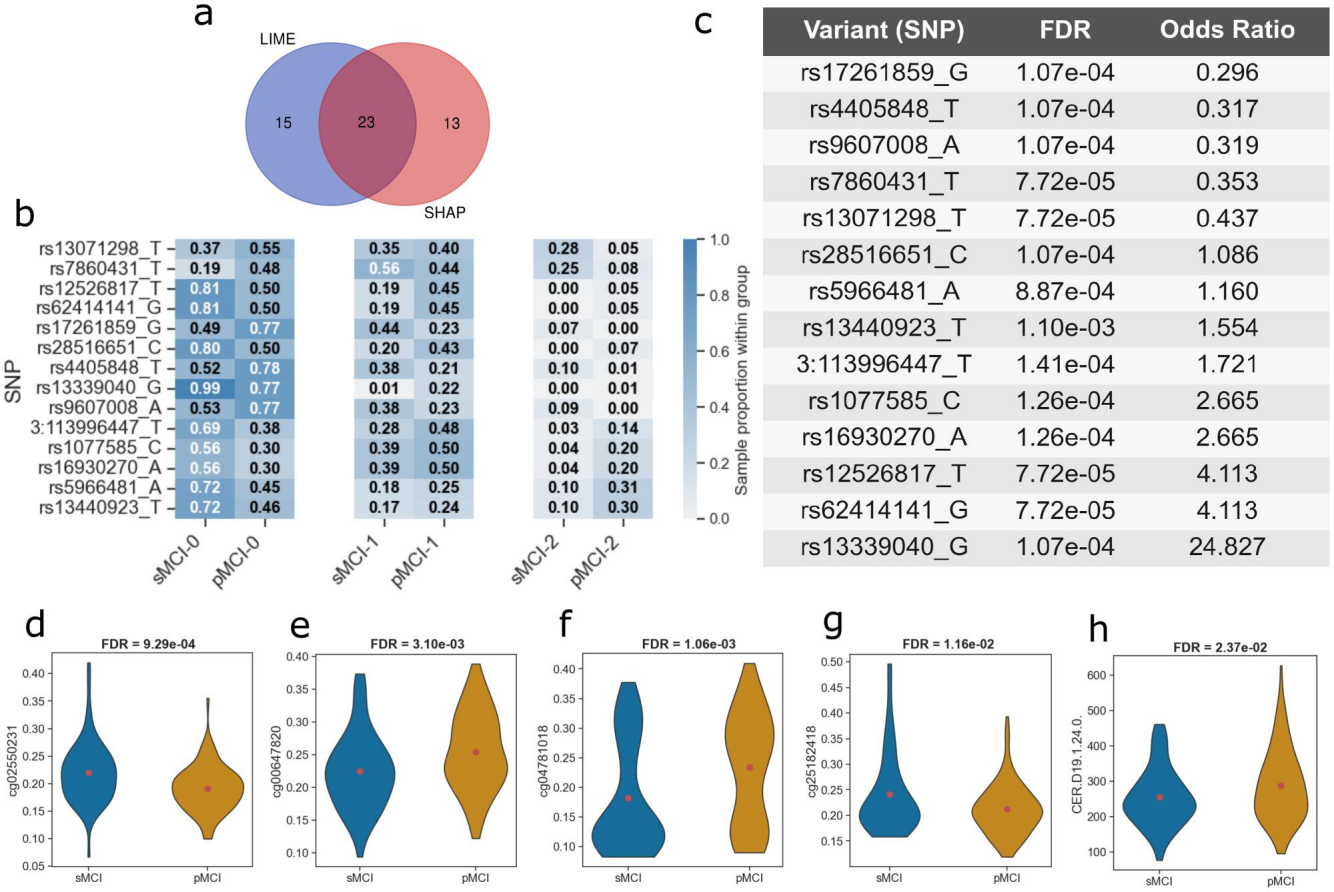
Feature importance and biomarker identification. (a) Venn diagram showing overlap between features consistently identified by LIME and SHAP across ten repeated runs. (b) Distribution of 14 significant SNPs across sMCI and pMCI participants, where 0, 1, and 2 indicate homozygous reference, heterozygous, and homozygous alternative configurations, respectively. (c) Table with significant variants, showing increasing risk of pMCI (OR>1) or are appearing protective (OR<1) (d–g) Violin plots depicting methylation levels of significant CpG sites with FDR values: (d) cg02550231 (*CDX2*), (e) cg00647820 (*DHX58*), (f) cg04781018 (*ZG16B*), and (g) cg25182418 (*TMC2*). (h) Violin plot of the lipid metabolite CER.D19.1.24.0, showing elevated abundance in pMCI relative to sMCI. All selected features were statistically significant (FDR<0.05).

Feature importance analysis using LIME and SHAP was conducted to identify biomarkers consistently contributing to the classification of sMCI and pMCI. The top 50 ranked features from each of the ten runs were compared, and features that appeared in all iterations were considered as important features. We compared important features reported by LIME and SHAP and found that 23 of them were reported by both methods (Fig. 3a). Among these 23 common important features, 14 SNPs, four DNA methylation sites, and one lipid metabolite were identified as statistically significant between sMCI and pMCI groups. Figure 3b shows the genotype distribution of the 14 significant SNPs across the two conditions, where 0, 1, and 2 represents homozygous reference, heterozygous, and homozygous alternative configurations, respectively. The corresponding table in Fig. 3c depicts their OR and significance, demonstrating clear allele-dependent differences between the two groups.

Across the 14 candidate variants, several SNPs were located near genes with functions relevant to neuronal maintenance, synaptic transmission, transcriptional regulation and extracellular matrix stability. Examples include *MAPK10/JNK3* (neuronal stress-kinase signaling), *SYN3* (synaptic vesicle priming), *FOXP1* and *TLE1* (transcriptional regulation), *COL10A1* (matrix remodeling), and the *MYH11/NDE1/ABCC1* region at 16p13.11, a CNV-sensitive locus with mechanistic links to amyloid-β transport and neurocognitive vulnerability (Krohn *et al.* 2011, Cuccaro *et al.* 2017). Additional SNPs were found near *SSPN* (12p11.2) and *NT5DC1* (6q22.1), which are not yet established AD-associated loci but map to genomic intervals recurrently detected in CNV-based studies of neurodegeneration, highlighting them as candidates for future investigation. These results do not assign causal roles to individual variants but instead suggest biologically coherent targets and under-explored genomic regions that may influence the transition from sMCI to pMCI.

The methylation loci that differed significantly between sMCI and pMCI are shown in Fig. 3d–g. Using the ADNI-provided annotation reference, each CpG site was mapped to its nearest associated gene. Two sites, cg02550231 (*CDX2*–Body (Island)) and cg25182418 (*TMC2*–Body(Island)), displayed higher methylation levels in sMCI, while cg00647820 (*DHX58*–Body(Island)) and cg04781018 (*ZG16B*–Promoter (TSS200)) were elevated in pMCI. These genes represent distinct biological domains, including innate immune signaling (*DHX58*), epithelial/goblet-cell differentiation programs (*CDX2*, *ZG16B*), and mechanotransduction and ion-channel activity (*TMC2*). Although their involvement in AD pathology has not been fully characterized, the observed methylation shifts are aligned with prior evidence indicating that coordinated methylation and transcriptional changes can help uncover novel AD-related mechanisms (Semick *et al.* 2019).

Finally, the lipid metabolite CER.D19.1.24.0 was elevated in pMCI relative to sMCI (Fig. 3h). This accords with evidence that circulating ceramides are altered in MCI and predict subsequent cognitive decline and hippocampal atrophy (Mielke *et al.* 2010). Together, these results show that the interpretable, repeatedly selected features from our multi-omics models cohere with biological processes linked to AD and merit targeted validation as blood-based markers of progression.

## Discussion

Our study demonstrates the potential of integrating blood-based multi-omics and demographic data to predict the conversion of MCI to AD. Using five ML classifiers and both early and late data integration strategies, we found that late integration significantly outperformed early integration for the L1-regularized logistic regression model. The L1-regularized logistic regression also achieved the highest predictive performance, with an F1 score of 90.7% on the test data when combining SNP and lipid modalities, outperforming recent models that rely on neuroimaging or cognitive assessment features.

Feature interpretability analysis using LIME and SHAP identified a reproducible subset of biomarkers significantly associated with MCI progression. These included 14 SNPs, four DNA methylation sites, and one lipid metabolite. Several of the associated genes, like *FOXP1*, *TLE1*, *COL10A1*, *MAPK10*, *MYH11*, and *SYN3*, have been implicated in AD-related pathways, particularly in synaptic regulation, neuroinflammation, and neuronal survival. Moreover, differential methylation at loci such as *CDX2*, *DHX58*, and *TMC2*, as well as elevated levels of ceramide metabolite CER.D19.1.24.0, align with known molecular mechanisms of neurodegeneration. These findings reinforce the biological relevance of the identified blood-based markers and highlight the capability of interpretable ML models to extract clinically meaningful insights from high-dimensional omics data. Although these biomarkers were not necessarily the top univariate associations within each omic layer, their consistent contribution in the multivariate predictive setting highlights them as hypothesis-generating candidates for future validation.

Despite these promising results, several limitations should be acknowledged. First, the dataset size was modest, particularly for multi-omics combinations, which may have restricted model generalizability and led to instability in the deep learning model. Second, while late integration improved overall performance and reduced overfitting, it also limits direct interpretability because the learned representations across omic layers are combined at a decision level rather than at the feature level. In contrast, early integration supports direct feature attribution but is more prone to overfitting given the high dimensionality of concatenated omics features. Future work should focus on developing hybrid or attention-based integration frameworks that balance interpretability and generalization by learning modality-specific representations while maintaining cross-omic interactions. Expanding the dataset size and including external validation cohorts will also be crucial for confirming the robustness and transferability of the identified biomarkers.

In summary, this work presents an interpretable, multi-omics-based framework that effectively distinguishes progressive from stable MCI using only peripheral blood biomarkers. By integrating statistical learning with biologically grounded interpretation, our approach provides a scalable and non-invasive foundation for early AD risk stratification and precision neurology.

Data used in preparation of this article were obtained from the ADNI database (https://adni.loni.usc.edu/). The investigators within ADNI contributed to the design and implementation of ADNI and/or provided data but did not participate in the analysis or writing of this report.

## Supplementary Material

vbag085_Supplementary_Data

## Data Availability

The data analyzed in this study were obtained from the ADNI database (https://adni.loni.usc.edu/) and are available to qualified researchers upon application.

## Code availability

The code used in this study, along with instructions and a toy dataset, is available at https://github.com/bozdaglab/AD_sMCI_vs_pMCI.
