# Supplementary material for: Predicting progression of Alzheimer’s disease using blood-based multi-omics data
Source: Bioinform Adv. 2026 Mar 28;6(1):vbag085. doi: 10.1093/bioadv/vbag085 (PMC13157226; doi:10.1093/bioadv/vbag085)
Supplement: vbag085_Supplementary_Data [file vbag085_supplementary_data.pdf]

Supplementary Information
Predicting progression of Alzheimer's disease using blood-based multi-omics data

Yashu Vashishath, Bizhan Alipour Pijani, Neha Goud Baddam,
Fahad Saeed, Serdar Bozdag and Alzheimer's Disease Neuroimaging Initiative

Table 1: Performance metrics for the L1 model. S denotes SNP, D denotes DNA methylation, G denotes gene expression, L denotes lipid, and B denotes bile acid modalities. Bold text indicates the highest F1 score, and underlined text indicates the second-highest score.

Model	Combination	Accuracy	Sensitivity	Specificity	Precision	F1 score
L1	B	59.75% ± 2.49	93.33% ± 9.25	9.38% ± 12.24	60.79% ± 1.64	73.42% ± 2.88
L1	B + D	59.5% ± 3.29	79.58% ± 4.99	29.38% ± 7.25	62.87% ± 2.26	70.18% ± 2.66
L1	B + G	61.75% ± 7.17	80.0% ± 8.96	34.38% ± 20.47	65.27% ± 6.44	71.51% ± 4.87
L1	B + G +D	62.75% ± 3.62	74.58% ± 5.36	45.0% ± 8.23	67.16% ± 3.28	70.57% ± 3.08
L1	D	57.5% ± 5.27	70.42% ± 7.72	38.12% ± 6.88	63.0% ± 3.65	66.4% ± 4.99
L1	G + D	58.75% ± 3.95	70.0% ± 6.75	41.88% ± 7.25	64.39% ± 2.76	66.95% ± 3.99
L1	G	59.5% ± 2.3	84.17% ± 14.41	22.5% ± 20.67	62.38% ± 2.78	70.99% ± 4.08
L1	L	60.0%0.0	96.67% ± 10.54	5.0% ± 15.81	60.67% ± 2.11	74.17% ± 2.64
L1	L + B	56.5% ± 6.99	78.33% ± 30.54	23.75% ± 33.31	54.99% ± 19.71	64.04% ± 22.88
L1	L + B + D	60.0% ± 3.33	78.33% ± 7.56	32.5% ± 11.33	63.67% ± 2.59	70.03% ± 3.26
L1	L + B + G	57.75% ± 5.95	74.17% ± 6.15	33.12% ± 11.43	62.61% ± 4.49	67.79% ± 4.38
L1	L + B + G + D	60.5% ± 3.87	73.75% ± 6.82	40.62% ± 6.75	65.08% ± 2.57	69.03% ± 3.82
L1	L + D	59.5% ± 5.11	70.42% ± 12.34	43.12% ± 18.27	66.14% ± 6.93	67.09% ± 6.6
L1	L + G	57.0% ± 5.63	75.83% ± 5.49	28.75% ± 15.92	61.93% ± 5.28	67.95% ± 3.31
L1	L + G + D	61.75% ± 3.92	77.08% ± 6.29	38.75% ± 10.12	65.5% ± 3.14	70.68% ± 3.4
L1	S	83.75% ± 3.39	89.58% ± 5.64	75.0% ± 7.22	84.49% ± 3.55	86.83% ± 2.94
L1	S + B	87.25% ± 2.75	95.42% ± 1.32	75.0% ± 7.22	85.28% ± 3.68	90.02% ± 1.97
L1	S + B + D	79.25% ± 3.34	89.17% ± 2.91	64.38% ± 8.36	79.15% ± 3.78	83.79% ± 2.31
L1	S + B + G	86.25% ± 4.89	91.67% ± 4.39	78.12% ± 10.31	86.58% ± 5.69	88.93% ± 3.85
L1	S + B + G + D	79.75% ± 5.33	92.08% ± 2.37	61.25% ± 13.11	78.48% ± 5.82	84.62% ± 3.55
L1	S + D	77.75% ± 2.99	92.08% ± 2.37	56.25% ± 8.33	76.1% ± 3.27	83.27% ± 1.9
L1	S + G	85.25% ± 2.99	92.92% ± 3.95	73.75% ± 10.54	84.55% ± 5.18	88.36% ± 2.07
L1	S + G + D	83.5% ± 3.76	92.5% ± 3.83	70.0% ± 6.45	82.3% ± 3.35	87.06% ± 2.92
L1	S + L	88.25% ± 4.09	95.0% ± 2.64	78.12% ± 8.96	86.91% ± 4.86	**90.71% ± 3.13**
L1	S + L + B	86.75% ± 3.13	93.33% ± 4.03	76.88% ± 7.25	85.99% ± 3.56	89.42% ± 2.5
L1	S + L + B + D	79.5% ± 4.53	88.75% ± 6.53	65.62% ± 9.88	79.72% ± 4.52	83.82% ± 3.72
L1	S + L + B + G	85.25% ± 2.49	93.33% ± 4.03	73.12% ± 7.25	84.08% ± 3.39	88.36% ± 1.91
L1	S + L + B + G + D	77.75% ± 3.22	91.25% ± 6.04	57.5% ± 9.22	76.53% ± 3.51	83.07% ± 2.62
L1	S + L + D	83.0% ± 4.05	90.83% ± 4.73	71.25% ± 7.91	82.72% ± 3.85	86.5% ± 3.26
L1	S + L + G	80.75% ± 5.66	88.75% ± 6.23	68.75% ± 8.33	81.09% ± 4.6	84.66% ± 4.56
L1	S + L + G + D	80.5% ± 2.84	92.92% ± 4.83	61.88% ± 8.04	78.68% ± 3.04	85.1% ± 2.25

Table 2: Performance metrics for the NN model. S denotes SNP, D denotes DNA methylation, G denotes gene expression, L denotes lipid, and B denotes bile acid modalities. Bold text indicates the highest F1 score, and underlined text indicates the second-highest score.

Model	Combination	Accuracy	Sensitivity	Specificity	Precision	F1 score
NN	B	53.0% ± 6.65	71.25% ± 25.87	25.62% ± 28.63	60.57% ± 7.69	62.04% ± 14.39
NN	B + D	57.0% ± 5.75	73.75% ± 19.25	31.87% ± 19.2	61.85% ± 2.79	65.94% ± 11.43
NN	B + G	51.5% ± 6.48	59.17% ± 12.23	40.0% ± 11.1	59.5% ± 4.97	58.94% ± 7.74
NN	B + G +D	53.75% ± 10.42	57.92% ± 22.61	47.5% ± 19.59	61.03% ± 12.05	57.92% ± 16.76
NN	D	53.25% ± 9.36	66.25% ± 41.83	33.75% ± 41.37	47.43% ± 25.31	53.87% ± 31.47
NN	G + D	54.0% ± 6.48	62.08% ± 24.25	41.88% ± 30.05	63.6% ± 7.54	59.29% ± 14.46
NN	G	56.5% ± 6.58	84.17% ± 21.05	15.0% ± 27.35	61.14% ± 7.57	68.69% ± 9.5
NN	L	54.0% ± 8.6	59.17% ± 29.38	46.25% ± 36.94	67.13% ± 15.81	56.98% ± 17.73
NN	L + B	48.25% ± 8.0	56.25% ± 21.72	36.25% ± 23.16	56.06% ± 6.96	54.39% ± 15.49
NN	L + B + D	53.5% ± 11.74	50.0% ± 25.76	58.75% ± 20.87	64.0% ± 12.75	53.19% ± 19.1
NN	L + B + G	49.0% ± 8.99	55.0% ± 21.05	40.0% ± 18.68	57.03% ± 7.73	54.68% ± 13.71
NN	L + B + G + D	50.75% ± 4.42	59.17% ± 15.57	38.12% ± 20.08	59.43% ± 5.05	58.21% ± 7.49
NN	L + D	59.25% ± 5.53	78.75% ± 12.65	30.0% ± 13.76	62.87% ± 3.3	69.52% ± 5.66
NN	L + G	58.25% ± 8.08	65.0% ± 22.24	48.12% ± 21.26	67.97% ± 12.22	62.94% ± 14.84
NN	L + G + D	60.0% ± 6.67	75.83% ± 14.67	36.25% ± 17.38	64.32% ± 5.14	68.97% ± 7.24
NN	S	55.0% ± 10.34	60.0% ± 29.48	47.5% ± 30.93	63.91% ± 7.23	57.56% ± 20.32
NN	S + B	62.75% ± 17.38	69.58% ± 36.43	52.5% ± 34.51	70.88% ± 19.71	63.68% ± 28.47
NN	S + B + D	60.5% ± 13.48	56.25% ± 25.02	66.88% ± 26.52	73.87% ± 20.35	60.55% ± 19.47
NN	S + B + G	62.75% ± 13.3	77.92% ± 15.96	40.0% ± 25.89	66.92% ± 11.72	<u>71.27% ± 11.06</u>
NN	S + B + G + D	64.0% ± 14.2	75.0% ± 17.12	47.5% ± 24.15	68.62% ± 11.68	71.03% ± 12.47
NN	S + D	53.5% ± 16.04	53.75% ± 35.16	53.12% ± 29.05	59.27% ± 27.82	51.34% ± 30.35
NN	S + G	60.25% ± 12.33	50.0% ± 30.49	75.62% ± 21.74	78.69% ± 12.46	54.47% ± 26.6
NN	S + G + D	61.25% ± 14.4	62.92% ± 26.82	58.75% ± 24.51	63.78% ± 24.9	62.52% ± 24.33
NN	S + L	70.25% ± 20.56	69.58% ± 33.85	71.25% ± 17.73	73.87% ± 21.0	68.96% ± 30.24
NN	S + L + B	68.25% ± 10.0	67.92% ± 25.54	68.75% ± 30.76	80.55% ± 10.9	69.8% ± 14.0
NN	S + L + B + D	67.25% ± 11.75	73.33% ± 26.37	58.13% ± 26.69	71.8% ± 11.45	69.69% ± 22.26
NN	S + L + B + G	64.0% ± 7.56	77.5% ± 19.27	43.75% ± 24.83	69.85% ± 11.75	71.05% ± 9.19
NN	S + L + B + G + D	63.5% ± 14.35	71.25% ± 23.61	51.88% ± 24.83	68.34% ± 12.55	68.05% ± 19.16
NN	S + L + D	66.0% ± 19.9	67.92% ± 28.06	63.12% ± 24.9	72.24% ± 25.3	68.22% ± 24.19
NN	S + L + G	68.0% ± 11.11	85.0% ± 19.56	42.5% ± 33.18	71.24% ± 11.55	**75.44% ± 10.83**
NN	S + L + G + D	59.0% ± 10.94	51.25% ± 28.33	70.62% ± 24.83	68.16% ± 27.12	55.39% ± 23.66

Table 3: Performance metrics for the RF model. S denotes SNP, D denotes DNA methylation, G denotes gene expression, L denotes lipid, and B denotes bile acid modalities. Bold text indicates the highest F1 score, and underlined text indicates the second-highest score.

Model	Combination	Accuracy	Sensitivity	Specificity	Precision	F1 score
RF	B	50.0% ± 4.25	56.25% ± 7.41	40.62% ± 9.88	58.73% ± 3.79	57.27% ± 4.99
RF	B + D	55.75% ± 7.55	66.25% ± 19.29	40.0% ± 17.97	62.4% ± 5.38	63.1% ± 10.09
RF	B + G	49.5% ± 6.75	67.08% ± 13.53	23.12% ± 12.17	56.48% ± 4.22	60.97% ± 7.43
RF	B + G +D	53.75% ± 4.45	78.33% ± 7.3	16.88% ± 10.64	58.63% ± 2.82	66.94% ± 3.5
RF	D	62.25% ± 3.62	78.75% ± 7.97	37.5% ± 7.22	65.39% ± 2.1	71.31% ± 3.91
RF	G + D	55.0% ± 6.67	58.75% ± 22.52	49.38% ± 21.13	63.93% ± 4.9	58.97% ± 12.81
RF	G	57.0% ± 5.5	83.75% ± 7.72	16.88% ± 7.82	60.14% ± 3.01	69.94% ± 4.45
RF	L	57.5% ± 5.77	67.08% ± 9.31	43.12% ± 10.81	63.97% ± 4.75	65.23% ± 5.88
RF	L + B	48.0% ± 7.53	55.0% ± 15.19	37.5% ± 18.4	56.93% ± 6.83	55.12% ± 9.57
RF	L + B + D	53.25% ± 6.57	62.92% ± 18.16	38.75% ± 25.31	61.87% ± 7.24	60.68% ± 9.06
RF	L + B + G	57.25% ± 7.31	75.83% ± 12.55	29.38% ± 19.33	62.1% ± 5.86	67.71% ± 6.97
RF	L + B + G + D	50.5% ± 8.4	46.67% ± 29.84	56.25% ± 29.17	65.18% ± 13.95	47.84% ± 22.24
RF	L + D	58.5% ± 6.37	68.33% ± 14.33	43.75% ± 16.67	65.09% ± 6.7	65.82% ± 7.59
RF	L + G	55.75% ± 7.55	68.75% ± 10.62	36.25% ± 16.08	62.06% ± 6.4	64.88% ± 6.78
RF	L + G + D	59.0% ± 4.28	77.5% ± 10.43	31.25% ± 18.87	63.36% ± 4.41	69.23% ± 3.59
RF	S	80.0% ± 5.14	91.67% ± 4.39	62.5% ± 6.59	78.58% ± 3.71	84.61% ± 3.95
RF	S + B	70.75% ± 10.68	89.17% ± 9.04	43.12% ± 17.79	70.49% ± 7.89	78.59% ± 7.64
RF	S + B + D	68.0% ± 12.95	86.67% ± 17.55	40.0% ± 18.45	68.23% ± 8.91	75.93% ± 11.75
RF	S + B + G	69.5% ± 5.99	83.33% ± 7.61	48.75% ± 19.5	71.8% ± 6.86	76.67% ± 3.82
RF	S + B + G + D	58.0% ± 7.15	71.25% ± 9.91	38.12% ± 17.54	63.81% ± 6.27	66.9% ± 6.05
RF	S + D	76.75% ± 10.14	79.58% ± 15.52	72.5% ± 16.46	81.61% ± 8.62	79.85% ± 10.82
RF	S + G	72.25% ± 10.77	80.83% ± 13.78	59.38% ± 7.93	74.44% ± 7.19	77.35% ± 10.16
RF	S + G + D	70.5% ± 10.19	80.0% ± 18.09	56.25% ± 24.47	74.42% ± 8.73	75.74% ± 10.62
RF	S + L	80.25% ± 2.99	92.5% ± 5.83	61.88% ± 10.81	78.79% ± 4.2	**84.88% ± 2.18**
RF	S + L + B	74.75% ± 7.02	85.0% ± 9.46	59.38% ± 15.66	76.45% ± 6.98	80.08% ± 5.61
RF	S + L + B + D	54.75% ± 11.02	63.33% ± 20.39	41.88% ± 10.23	60.76% ± 8.3	61.28% ± 14.13
RF	S + L + B + G	66.0% ± 5.8	87.5% ± 7.86	33.75% ± 20.24	67.14% ± 5.96	75.58% ± 3.37
RF	S + L + B + G + D	76.25% ± 11.86	87.92% ± 15.52	58.75% ± 11.49	75.77% ± 8.69	81.13% ± 11.44
RF	S + L + D	73.5% ± 9.22	85.83% ± 18.02	55.0% ± 20.37	75.14% ± 7.44	78.67% ± 10.77
RF	S + L + G	74.5% ± 3.29	92.92% ± 3.43	46.88% ± 8.96	72.54% ± 2.94	81.41% ± 2.08
RF	S + L + G + D	59.5% ± 7.34	65.83% ± 24.12	50.0% ± 27.95	68.41% ± 7.14	64.09% ± 11.87

Table 4: Performance metrics for the SVM model. S denotes SNP, D denotes DNA methylation, G denotes gene expression, L denotes lipid, and B denotes bile acid modalities. Bold text indicates the highest F1 score, and underlined text indicates the second-highest score.

Model	Combination	Accuracy	Sensitivity	Specificity	Precision	F1 score
SVM	B	59.5% ± 1.05	87.92% ± 19.88	16.88% ± 27.8	62.28% ± 3.99	71.41% ± 6.07
SVM	B + D	58.0% ± 3.69	62.08% ± 9.1	51.88% ± 9.34	66.01% ± 2.76	63.65% ± 5.29
SVM	B + G	55.75% ± 8.58	73.33% ± 12.14	29.38% ± 11.8	60.72% ± 6.04	66.22% ± 8.22
SVM	B + G +D	55.0% ± 3.12	60.0% ± 5.27	47.5% ± 11.1	63.57% ± 4.62	61.47% ± 2.68
SVM	D	58.75% ± 3.95	72.08% ± 6.23	38.75% ± 9.68	63.97% ± 3.24	67.63% ± 3.52
SVM	G + D	58.0% ± 3.07	60.42% ± 5.97	54.37% ± 6.62	66.58% ± 2.73	63.2% ± 3.75
SVM	G	58.75% ± 5.8	87.5% ± 14.16	15.62% ± 20.25	61.25% ± 5.13	71.48% ± 5.46
SVM	L	59.25% ± 2.37	96.67% ± 10.54	3.12% ± 9.88	59.93% ± 0.23	73.77% ± 3.88
SVM	L + B	64.75% ± 4.63	81.25% ± 10.25	40.0% ± 13.57	67.33% ± 4.37	73.25% ± 4.37
SVM	L + B + D	57.5% ± 5.89	62.5% ± 9.62	50.0% ± 14.43	65.54% ± 6.08	63.62% ± 5.98
SVM	L + B + G	61.25% ± 5.3	78.75% ± 8.44	35.0% ± 8.94	64.53% ± 3.43	70.79% ± 4.62
SVM	L + B + G + D	55.75% ± 4.09	58.33% ± 8.1	51.88% ± 8.86	64.63% ± 3.51	61.04% ± 5.06
SVM	L + D	54.5% ± 4.05	53.33% ± 9.58	56.25% ± 15.87	65.77% ± 7.24	58.07% ± 5.35
SVM	L + G	60.5% ± 4.68	75.0% ± 9.21	38.75% ± 10.12	64.84% ± 3.71	69.3% ± 4.92
SVM	L + G + D	58.75% ± 2.95	67.92% ± 10.77	45.0% ± 11.33	65.14% ± 2.15	66.01% ± 5.15
SVM	S	81.5% ± 3.76	90.83% ± 3.83	67.5% ± 8.23	80.92% ± 4.28	85.51% ± 2.87
SVM	S + B	84.0% ± 3.37	90.42% ± 4.41	74.38% ± 8.04	84.32% ± 4.01	<u>87.15% ± 2.66</u>
SVM	S + B + D	71.25% ± 7.75	72.5% ± 14.72	69.38% ± 9.97	78.15% ± 4.77	74.54% ± 8.44
SVM	S + B + G	83.75% ± 3.17	89.17% ± 3.51	75.62% ± 6.88	84.73% ± 3.7	86.82% ± 2.51
SVM	S + B + G + D	74.0% ± 7.09	78.33% ± 11.59	67.5% ± 11.33	78.54% ± 5.49	78.01% ± 7.27
SVM	S + D	72.0% ± 4.68	72.5% ± 9.66	71.25% ± 7.34	79.3% ± 3.91	75.36% ± 5.66
SVM	S + G	81.75% ± 2.06	86.67% ± 3.83	74.38% ± 6.88	83.74% ± 3.51	85.07% ± 1.63
SVM	S + G + D	70.75% ± 5.53	77.92% ± 7.1	60.0% ± 11.1	74.82% ± 6.11	76.12% ± 4.77
SVM	S + L	84.25% ± 4.87	90.0% ± 3.51	75.62% ± 9.06	84.89% ± 5.06	**87.32% ± 3.75**
SVM	S + L + B	84.0% ± 4.74	88.75% ± 6.53	76.88% ± 8.86	85.43% ± 4.6	86.89% ± 4.07
SVM	S + L + B + D	77.5% ± 4.56	80.0% ± 10.54	73.75% ± 9.22	82.46% ± 4.29	80.72% ± 5.35
SVM	S + L + B + G	81.5% ± 4.89	90.0% ± 5.62	68.75% ± 7.8	81.3% ± 4.01	85.35% ± 3.99
SVM	S + L + B + G + D	72.5% ± 4.86	78.33% ± 8.96	63.75% ± 13.11	76.89% ± 5.03	77.23% ± 4.54
SVM	S + L + D	81.0% ± 4.44	86.25% ± 6.82	73.12% ± 7.25	82.95% ± 3.98	84.41% ± 3.94
SVM	S + L + G	81.75% ± 4.57	93.33% ± 2.15	64.38% ± 10.23	79.94% ± 4.63	86.06% ± 3.1
SVM	S + L + G + D	76.0% ± 4.89	82.08% ± 6.53	66.88% ± 8.86	78.97% ± 4.38	80.35% ± 4.17

Table 5: Performance metrics for the XG-Boost model. S denotes SNP, D denotes DNA methylation, G denotes gene expression, L denotes lipid, and B denotes bile acid modalities. Bold text indicates the highest F1 score, and underlined text indicates the second-highest score.

Model	Combination	Accuracy	Sensitivity	Specificity	Precision	F1 score
XG-Boost	B	53.75% ± 5.17	58.75% ± 6.65	46.25% ± 9.86	62.27% ± 5.1	60.29% ± 4.9
XG-Boost	B + D	52.5% ± 5.77	56.67% ± 15.86	46.25% ± 21.08	62.13% ± 6.54	57.88% ± 9.21
XG-Boost	B + G	52.25% ± 6.29	68.33% ± 13.35	28.12% ± 12.59	58.59% ± 4.05	62.67% ± 7.78
XG-Boost	B + G +D	51.5% ± 10.08	47.92% ± 25.63	56.88% ± 18.97	56.54% ± 21.11	50.36% ± 22.66
XG-Boost	D	51.75% ± 7.91	63.33% ± 9.78	34.38% ± 11.51	59.11% ± 5.98	60.99% ± 7.09
XG-Boost	G + D	57.25% ± 7.59	60.0% ± 19.76	53.12% ± 25.39	66.92% ± 7.07	61.28% ± 11.57
XG-Boost	G	53.75% ± 5.03	71.25% ± 10.1	27.5% ± 8.44	59.47% ± 2.86	64.62% ± 5.69
XG-Boost	L	58.0% ± 4.97	73.33% ± 5.27	35.0% ± 7.91	62.89% ± 3.58	67.66% ± 3.97
XG-Boost	L + B	53.25% ± 7.27	62.08% ± 19.09	40.0% ± 22.48	60.85% ± 6.63	60.16% ± 11.38
XG-Boost	L + B + D	54.75% ± 6.17	55.42% ± 16.44	53.75% ± 20.45	65.12% ± 6.77	58.48% ± 9.35
XG-Boost	L + B + G	58.25% ± 5.53	74.17% ± 10.54	34.38% ± 20.25	63.6% ± 5.74	67.89% ± 4.66
XG-Boost	L + B + G + D	56.0% ± 6.58	66.25% ± 7.2	40.62% ± 15.1	63.02% ± 6.12	64.34% ± 5.0
XG-Boost	L + D	57.0% ± 4.22	70.0% ± 14.27	37.5% ± 16.67	63.01% ± 3.78	65.58% ± 6.16
XG-Boost	L + G	55.5% ± 6.43	70.42% ± 13.67	33.12% ± 8.86	60.95% ± 3.71	64.99% ± 7.59
XG-Boost	L + G + D	53.25% ± 9.06	51.67% ± 19.27	55.62% ± 26.43	65.1% ± 12.3	55.64% ± 12.1
XG-Boost	S	74.0% ± 8.27	82.08% ± 7.36	61.88% ± 12.66	76.56% ± 7.31	79.13% ± 6.67
XG-Boost	S + B	77.25% ± 7.5	85.83% ± 6.27	64.38% ± 20.84	79.4% ± 8.5	82.09% ± 4.97
XG-Boost	S + B + D	78.75% ± 9.88	84.17% ± 7.3	70.62% ± 22.64	82.49% ± 11.27	82.86% ± 7.15
XG-Boost	S + B + G	76.0% ± 11.68	76.67% ± 14.86	75.0% ± 10.62	81.68% ± 9.52	78.79% ± 11.9
XG-Boost	S + B + G + D	74.0% ± 6.37	75.83% ± 10.54	71.25% ± 16.46	80.98% ± 8.78	77.57% ± 6.47
XG-Boost	S + D	76.5% ± 8.35	87.5% ± 6.21	60.0% ± 25.21	78.32% ± 10.56	82.02% ± 5.0
XG-Boost	S + G	79.0% ± 6.26	82.5% ± 9.38	73.75% ± 14.37	83.24% ± 7.27	82.39% ± 5.61
XG-Boost	S + G + D	68.0% ± 10.12	72.92% ± 13.21	60.62% ± 18.65	74.17% ± 9.24	72.92% ± 9.28
XG-Boost	S + L	76.25% ± 7.93	88.75% ± 5.22	57.5% ± 21.41	76.96% ± 9.47	82.0% ± 4.99
XG-Boost	S + L + B	80.25% ± 8.29	86.25% ± 6.82	71.25% ± 17.97	82.79% ± 10.1	84.13% ± 6.25
XG-Boost	S + L + B + D	71.75% ± 15.32	70.42% ± 23.77	73.75% ± 23.16	80.94% ± 16.69	73.06% ± 19.22
XG-Boost	S + L + B + G	76.25% ± 5.43	90.42% ± 3.95	55.0% ± 14.07	75.48% ± 5.46	82.13% ± 3.5
XG-Boost	S + L + B + G + D	68.5% ± 13.19	67.92% ± 24.3	69.38% ± 26.1	78.69% ± 10.13	69.85% ± 18.11
XG-Boost	S + L + D	78.0% ± 6.85	80.0% ± 7.56	75.0% ± 16.67	83.86% ± 9.43	81.42% ± 5.24
XG-Boost	S + L + G	81.0% ± 5.92	86.67% ± 9.38	72.5% ± 15.65	83.61% ± 8.59	**84.48% ± 4.96**
XG-Boost	S + L + G + D	81.25% ± 6.9	82.08% ± 12.27	80.0% ± 15.81	87.21% ± 8.61	83.76% ± 6.47
